# Prevalence of epilepsy in China between 1990 and 2015: A systematic review and meta–analysis

**DOI:** 10.7189/jogh.07-020706

**Published:** 2017-12

**Authors:** Peige Song, Yezhou Liu, Xinwei Yu, Jingjing Wu, Adrienne N Poon, Alessandro Demaio, Wei Wang, Igor Rudan, Kit Yee Chan, on behalf of the Global Health Epidemiology Research Group (GHERG)

**Affiliations:** 1 Centre for Global Health Research, Usher Institute of Population Health Sciences and Informatics, University of Edinburgh, Scotland, UK; 2 Beijing Municipal Key Laboratory of Clinical Epidemiology, School of Public Health, Capital Medical University, Beijing, China; 3 Xijing Hospital of Digestive Diseases, the Fourth Military Medical University; 4 School of Medical and Health Sciences, Edith Cowan University, Perth, Western Australia, Australia; 5 Beijing Tiantan Hospital, Capital Medical University, Beijing, China; 6 Department of Internal Medicine, School of Medicine & Health Sciences, The George Washington University, Washington, D.C., USA; 7 Harvard Global Equity Initiative, Harvard Medical School, Boston, Massachusetts, USA; 8 Copenhagen School of Global Health, University of Copenhagen, Denmark; 9 Nossal Institute for Global Health, University of Melbourne, Australia; * Joint–first authors; ** Joint–last authors

## Abstract

**Background:**

Epilepsy is a major neurological disorder that affects approximately 65 million people worldwide. Globally, the burden of epilepsy is not evenly distributed, with more than 80% of sufferers residing in low– and middle–income countries. This study estimates the burden of epilepsy in mainland China from 1990 to 2015 and explores the variations of burden by age and gender.

**Methods:**

We conducted a systematic review of the peer–reviewed literature from 1990 to 2015 using Chinese and English academic databases (CNKI, WanFang, VIP and PubMed) to identify population–based prospective studies on the prevalence of epilepsy in mainland Chinese. Multilevel mixed–effects logistic regression was used to estimate the prevalence of lifetime epilepsy (LTE), and restricted cubic regression splines were applied to model the functional forms of the non–linear effects of age and LTE prevalence. Random–effect meta–analysis was used to obtain the pooled prevalence of 1–year active epilepsy (AE), 2–year AE and 5–year AE separately. To estimate the number of people with LTE and AE in the years 1990, 2000, and 2015, LTE and AE prevalence were multiplied by the total population of mainland China of the corresponding year.

**Findings:**

Analyses were conducted using 39 prevalence studies that met the inclusion criteria and comprised 77 separate data points (37 on LTE, 16 on 1–year AE, 12 on 2–year AE and 12 on 5–year AE). In 1990, the prevalence of LTE ranged from 1.31‰ (95% CI = 0.85–2.00) in the 0–4 age group to 2.42‰ (95% confidence interval CI = 1.60–3.65) in the 30–34 age group. By 2015, the LTE prevalence had increased to 4.57‰ (95% CI = 2.52–8.27) in the 0–4 group and 8.43‰ (95% CI = 4.71–15.04) in the 30–34 group. Over the 25–year period, the overall prevalence of LTE had steadily increased by 259%, from 1.99‰ (95% CI = 1.31–3.02) in 1990 to 7.15‰ (95% CI = 3.98–12.82) in 2015. The rates of increase were similar across the whole age spectrum, fluctuating around 250%. Between 1990 and 2015, the total number of people with LTE in mainland China increased by 328%, from 2.30 million (95% CI = 1.51–3.49) in 1990 to 9.84 million (95% CI = 5.48–17.64) in 2015. The pooled 1–year, 2–year, and 5–year AE prevalence were 3.79‰ (95% CI = 3.31–4.34), 4.08‰ (95% CI = 3.41–4.89) and 4.19‰ (95% CI = 3.42–5.15).

**Conclusions:**

The burden of LTE in China has increased substantially between 1990 and 2015, with the prevalence of LTE having more than doubled and the number of people with LTE more than tripled. The large amount of AE cases in China calls for optimal management and treatment. More high–quality epidemiological studies on LTE and AE prevalence are still needed.

Epilepsy is a disorder of neuronal excitability, characterised predominantly by unpredictable and recurrent seizures of cerebral origin [1]. As one major neurological disorder, epilepsy affects approximately 65 million people worldwide, ranging from neonates to elderly [2–5]. The burden of epilepsy is not only limited in neurological deficits but also includes devastating psychological and psychiatric problems [6,7], influencing the quality of personal, familial and social life significantly [6,8,9]. If left untreated, epilepsy would be incapacitating and sometimes fatal. People living with epilepsy generally have higher disability and mortality rates [5,10,11]. Worldwide, it is estimated that mortality in people with epilepsy is two to three times higher than in the general population [12], while the Global Burden of Disease (GBD) study for 2013 estimated the disability–adjusted life year for epilepsy to be 253 per 100 000 people [13,14]. Although effective and cost–effective medications exist for controlling seizures, many people with epilepsy are excluded from treatments due to cultural, economic and other factors [11,15]. The global burden of epilepsy is not distributed evenly, with more than 80% of people with epilepsy residing in low– and middle–income countries (LMICs) [4,16], where the majority of people with epilepsy receive inadequate treatment and management [9,16].

Quality epidemiological data are crucial for estimating the burden of epilepsy which in turn serve to inform policies on resource allocation and disease management [17,18]. Over the past decades, the number of prevalence studies on epilepsy has grown considerably, making it possible to synthesise prevalence of epilepsy at a regional and global level [10,19–24]. Such estimates are valuable despite potential confounders that arise with the difference in sampling, case definitions, case ascertainments, and screening tools [10,21,23]. The prevalence estimates of epilepsy in developed countries are generally consistent with each other, whereas those in developing countries are often made for isolated geographical areas and they vary widely [10,22,23]. It is estimated that the prevalence of epilepsy in LMICs is twice as high as in high–income countries (HICs), making it an even more important global health issue in these settings [16,25].

Nevertheless, the reported prevalence of epilepsy is likely to be conservative, because underdiagnosis and misdiagnosis are common in resource–poor areas [10,25]. Furthermore, as a disease marked with stigma and prejudice across the world and throughout history, epilepsy may be largely concealed because of profound cultural and social restrictions [7,8,26]. The number of high–quality studies regarding the epidemiology of epilepsy in the developing world is quite small, which makes the estimate of epilepsy prevalence very difficult [10].

In China, although substantial economic development and improvement of health services occurred in the past decades, the diversity of development and demographic structures across the whole country still limits the opportunity for an estimate of epilepsy prevalence at the national level [19,27]. However, the large volume of data on the prevalence of epilepsy in Chinese bibliographical databases makes it feasible to explore the burden of epilepsy from a modelling approach [28,29]. For instance, Lian Gu and colleagues revealed an overall epilepsy prevalence of 2.89‰ in Mainland China by using the meta–analysis method [19]. However, no temporal trend has been analysed in their study, and the prevalence estimates were not conducted in a large age span, which may have limited their study to some extent. Moreover, as new evidence continues to emerge, such evidence synthesis should ideally be updated using a more detailed approach. For these reasons, we conducted a systematic review of the literature in both Chinese and English databases to analyse the temporal distribution of epilepsy prevalence in China from 1990 to 2015. We also investigated the variations in prevalence by age and gender.

## METHODS

### Literature search

We conducted a parallel systematic review of the published literature from 1990 to 2015 using PubMed and three Chinese databases; China National Knowledge Infrastructure (CNKI), Wanfang Data, VIP in accordance with the Preferred Reporting Items for Systematic Reviews and Meta–Analyses (PRISMA) Guidelines and the Guidelines for Accurate and Transparent Health Estimates Reporting (GATHER) statement [30,31]. The search strategy for PubMed was ((epilepsy) AND (China OR Chinese) AND (inciden* OR prevalen* OR morbidity OR mortality)). The search terms for the Chinese databases were the term epilepsy in both China and English, and two versions of Chinese terms for ‘incidence’ and ‘prevalence’, attack rate, mortality, fatality, epi*, burden, epidemiological survey and cross–sectional investigation. The precise combination of the search term for the four databases search is detailed in Table S1 in the **Online Supplementary Document(Online Supplementary Document)**.

### Selection criteria

Our inclusion criteria were: (1) population–based studies; (2) studies of mainland Chinese populations in mainland China; (3) studies that provide prevalence of lifetime epilepsy (LTE) and/or active epilepsy (AE); (4) studies that include clear case definitions (the case definitions used to define a case of epilepsy are described in detail in Table S2 in **Online Supplementary Document(Online Supplementary Document)**). Our exclusion criteria were: (1) case–control and hospital–based studies; (2) studies of populations outside of mainland China (including Hong Kong and Taiwan); (3) studies of specific areas; eg, mining or fishery districts; and (4) reviews and conference abstracts; (5) duplicate publications; (6) studies that included febrile convulsions and provoked seizures in their estimates of epilepsy prevalence; (7) studies with unclear case definitions; and (8) studies with inconsistent results.

### Data extraction

Data were independently extracted (PS, YZ and XY for data in Chinese; PS, KYC and AP for data in English). A database was set up to record the extracted information which included names of authors, published year, study setting (urban and/or rural), medium year of data collection, sampling method, case definition, sample size, number of epilepsy cases, and epilepsy prevalence.

In epidemiological studies of epilepsy, the prevalence estimates of LTE and AE are generally reported separately, where the LTE prevalence is the proportion of individuals manifesting a disorder anytime during the earlier period of their life up to the point of investigation [32], and AE prevalence represents the proportion of individuals who have experienced at least two unprovoked seizures within a certain period of time (1 year, 2 years, and 5 years) up to the point of investigation [2,10,33,34]. In data extraction process, prevalence estimates were classified into these two exclusive groups based on the definitions or investigation methodologies provided in each study.

### Statistical analysis

Our analysis of epilepsy prevalence was conducted for LTE, 1–year AE, 2–year AE and 5–year AE separately. For studies that provided estimates of LTE prevalence, multiple data points were available in each study to contribute to the overall database. To take into account the availability of different data points from the same study, meta–analysis via multilevel mixed–effects models was adopted [35]. To investigate whether LTE prevalence varied significantly according to different demographic factors (age and gender) or had a secular trend (study year), univariate meta–regression was adopted to test their significance consecutively. Restricted cubic regression splines were used to model the functional forms of the non–linear effects of age and LTE prevalence. Variables that significantly correlated with LTE prevalence in the univariate analyses were then included in the final multivariate regression model. Due to the paucity of studies that reported AE prevalence, effects of demographic factors and secular trends could not be explored using meta–regression. Instead, random–effect meta–analysis (DerSimonian Laird method) was applied to obtain the pooled prevalence [36].

To estimate the number of people with LTE and AE in the years 1990, 2000 and 2015, LTE and AE prevalence were multiplied by the population of China in the corresponding years using population data from the United Nations Population Division (UNPD) [37]. All the analyses were conducted in R v3.3.0 (R Development Core Team; http://www.R–project.org).

## RESULTS

### Systematic review

**Figure 1:**
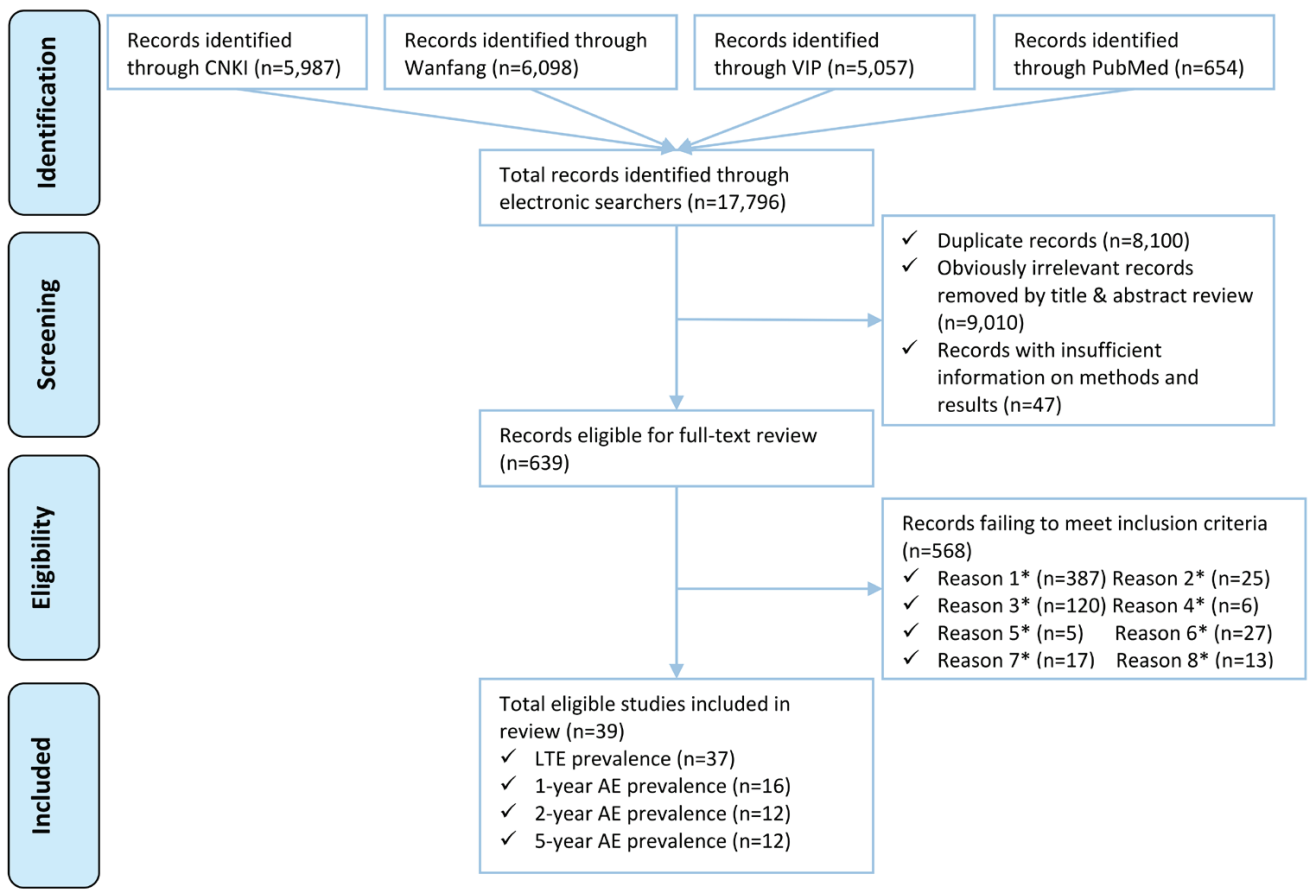
PRISMA flow diagram. *Note: *Reason 1–Papers that were not population–based epilepsy study; *Reason 2–Studies that were not based in Mainland China; *Reason 3–Papers with no numerical prevalence measure of epilepsy; *Reason 4–Studies with no clear time period; *Reason 5–Studies only reported febrile convulsions or provoked seizures; *Reason 6–Multiple publications of the same study; *Reason 7–Studies only reported specific subtype of epilepsy; *Reason 8–Unclear prevalence type.

Our database searches returned 17 796 titles. After removing 8100 duplicates, and 9010 titles and abstracts that contained no information on epilepsy prevalence, and 47 papers with insufficient information on methods and results, 639 full–text papers were reviewed. Of the 39 full–text papers that met our inclusion criteria, 37 reported LTE prevalence, 16 reported 1–year AE prevalence, 12 reported 2–year AE prevalence and 12 reported 5–year AE prevalence (**Figure 1**).

### Study characteristics

**Table 1** summarises the key characteristics of the 39 studies. All studies were cross–sectional in design. Most of them were large studies published after 2000. Participants were typically investigated by neurologists using standard international questionnaires (eg, by World Health Organization [WHO] or International Community–based Epilepsy Research Group [ICBERG]). For more details of the studies, see Table S2 in the **Online Supplementary Document(Online Supplementary Document)**.

**Table 1:** Main characteristics of the retained studies

Characteristics of study	Studies with LTE prevalence (n = 37, %)	Studies with 1–year AE prevalence (n = 16, %)	Studies with 2–year AE prevalence (n = 12, %)	Studies with 5–year AE prevalence (n = 12, %)
**Year published:**				
1990–1999	12 (32.4)	0 (0.0)	0 (0.0)	0 (0.0)
2000–2009	16 (43.2)	11 (68.8)	8 (66.7)	7 (58.3)
2010–2016	9 (24.3)	5 (31.3)	4 (33.3)	5 (41.7)
**Setting:**				
Urban	4 (10.8)	1 (6.3)	1 (8.3)	1 (8.3)
Rural	21 (56.8)	14 (87.5)	10 (83.3)	10 (83.3)
Mixed	9 (24.3)	1 (6.3)	1 (8.3)	1 (8.3)
Both	3 (8.1)	0 (0.0)	0 (0.0)	0 (0.0)
**Sample size:**				
4000–10 000	9 (24.3)	2 (12.5)	1 (8.3)	1 (8.3)
10 001–50 000	17 (45.9)	11 (68.8)	9 (75.0)	10 (83.3)
50 001–200 000	8 (21.6)	2 (12.5)	2 (16.7)	1 (8.3)
200 001–900 000	3 (8.1)	1 (6.3)	0 (0.0)	0 (0.0)
**Screening tool:**				
WHO questionnaire	14 (37.8)	9 (56.3)	7 (58.3)	7 (58.3)
ICBERG questionnaire	5 (13.5)	4 (25.0)	4 (33.3)	4 (33.3)
Self–designed questionnaire	10 (27)	1 (6.3)	0 (0.0)	0 (0.0)
Questionnaire based on ILAE/CMA/ BNI diagnosis	7 (18.9)	2 (12.5)	1 (8.3)	1 (8.3)
Not specified	1 (2.7)	0 (0.0)	0 (0.0)	0 (0.0)
**Diagnosis of epilepsy:**				
By neurologists	27 (73.0)	12 (75.0)	9 (75.0)	10 (83.3)
By trained physicians	6 (16.2)	3 (18.8)	2 (16.7)	2 (16.7)
By trained investigators	2 (5.4)	0 (0.0)	0 (0.0)	0 (0.0)
Not specified	2 (5.4)	1 (6.3)	1 (8.3)	0 (0.0)

LTE – lifetime epilepsy, AE – active epilepsy, WHO – World Health Organization, ICBERG – International Community–based Epilepsy Research Group, ILAE – International League Against Epilepsy, CMA – Chinese Medical Association, BNI – Beijing Neurosurgical Institute

### Estimates of LTE prevalence and number of cases in China

**Figure 2:**
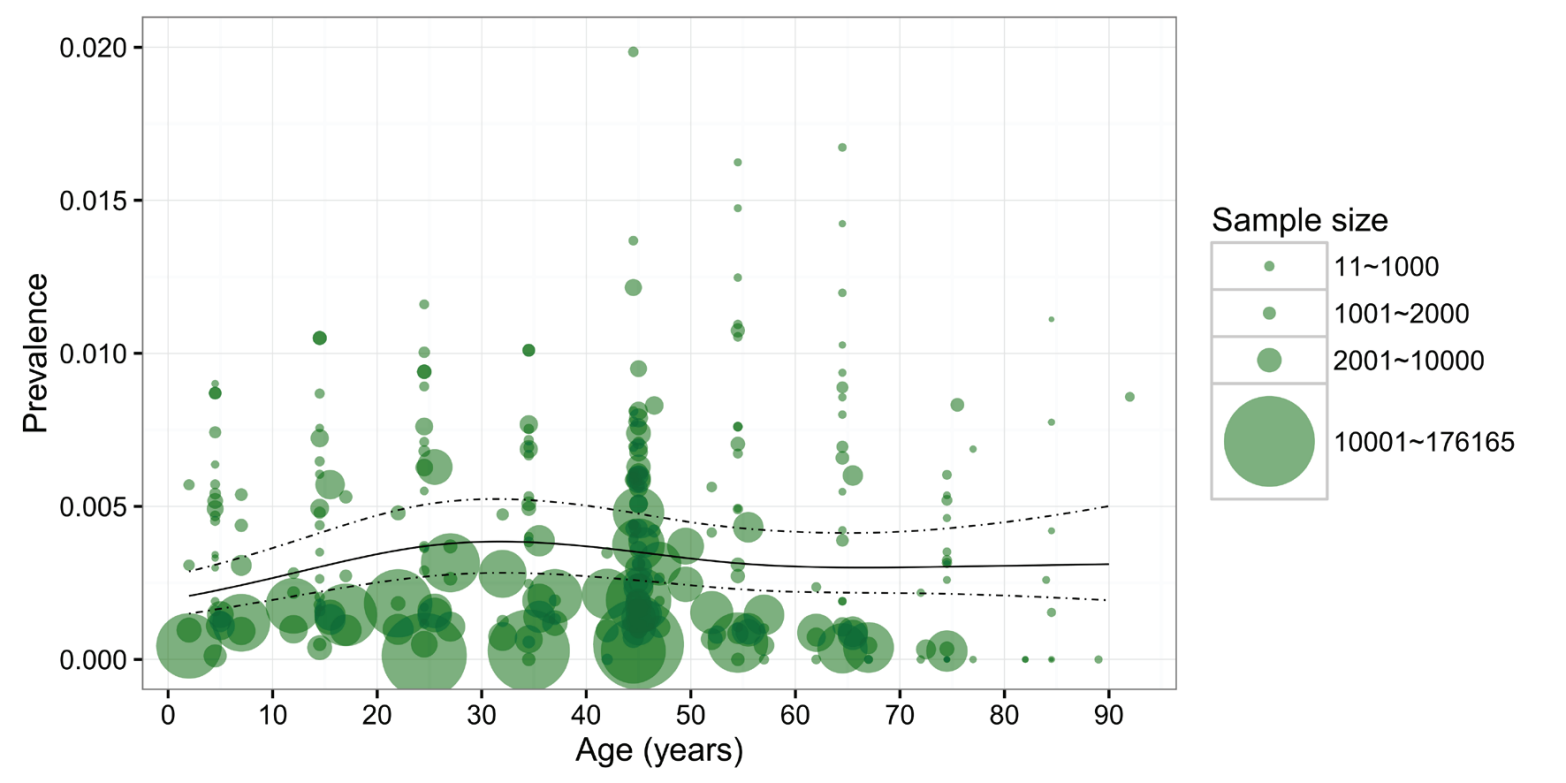
Age–specific prevalence of lifetime epilepsy (LTE) in China based on the data points from the included studies. Note: The size of each bubble is proportional to the sample size, the regression lines are based on the retained data points.

The 37 studies that reported LTE prevalence involved a combined total of 2 851 219 participants. Of these, 5813 met the criteria for LTE diagnosis, giving an LTE prevalence of 2.04‰. A total of 274 specific data points based on age, gender and location provided the information on LTE prevalence. Based on these informative data points, the gender–specific relationship between age and LTE prevalence was explored and it is shown in **Figure 2**. Generally, the larger studies yielded lower LTE prevalence in both males and females across most of the age spectrum.

In the univariate meta–regression analysis (Table S3 in **Online Supplementary Document(Online Supplementary Document)**), no gender difference was found in LTE prevalence, whereas age and study year were all significantly associated with LTE prevalence. The final formula generated from the multilevel mixed–effects meta–analysis is shown below:  

Where:

odds = p/(1–p), p indicates the prevalence of LTE

year = calendar year

age1–age4 are variables created in the process of fitting cubic splines (knots: 4.5, 24.5, 45.0, 54.5, 74.5)

**Figure 3:**
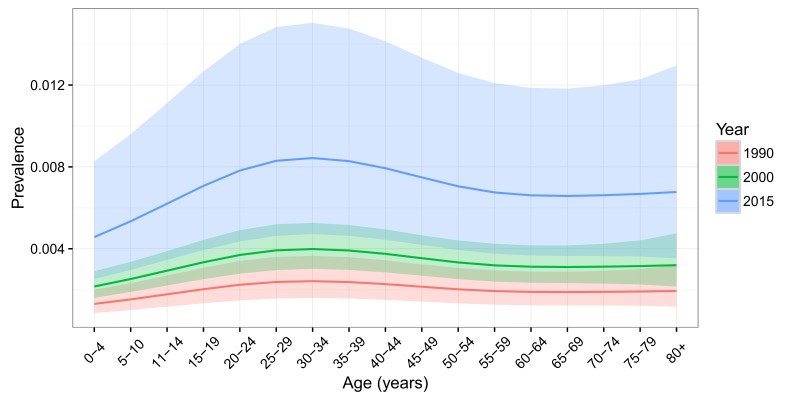
Age–specific prevalence of lifetime epilepsy (LTE) in China in the years 1990, 2000 and 2015, with 95% confidence intervals.

Based on the final regression model, age–specific LTE prevalence in mainland China was calculated for the years 1990, 2000 and 2015 (**Table 2** and **Figure 3****)**. In 1990, the prevalence of LTE was lowest in the 0–4 age group (1.31‰; 95% CI = 0.85–2.00) and highest in the 30–34 age group (2.42‰; 95% CI:1.60–3.65). By 2015, this prevalence had increased by three–fold to 4.57‰ (95% CI = 2.52–8.27) in the 0–4 age group and 8.43‰ in the 30–34 age group (95% CI = 4.71–15.04). Over 25 years, the overall LTE prevalence had steadily increased by 259%, from 1.99‰ (95% CI = 1.31–3.02) in 1990 to 7.15‰ (95% CI = 3.98–12.82) in 2015. This rate of increase is similar across the entire age spectrum, fluctuating around 250%.

**Table 2:** Estimated age–specific prevalence of and numbers of people with lifetime epilepsy (LTE) in China in the years 1990, 2000 and 2015, and the rate of change from 1990 to 2015 by age group

	Prevalence of LTE (‰)			Number of LTE cases (thousands)			Rate of change (1990–2015) for:	
**Age (years)**	**1990**	**2000**	**2015**	**1990**	**2000**	**2015**	**Prevalence (in %)**	**Number of cases (in %)**
0–4	1.31 (0.85–2.00)	2.16 (1.60–2.90)	4.57 (2.52–8.27)	173.05 (113.00–264.94)	172.13 (127.74–231.91)	380.15 (209.75–687.84)	+250	+119
5–9	1.53 (1.00–2.32)	2.52 (1.89–3.36)	5.34 (2.96–9.60)	155.95 (102.60–236.98)	270.71 (203.15–360.67)	419.97 (233.14–755.10)	+250	+169
10–14	1.77 (1.17–2.69)	2.93 (2.21–3.88)	6.20 (3.45–11.12)	174.30 (114.97–264.15)	383.76 (289.21–509.10)	466.92 (259.90–837.01)	+250	+168
15–19	2.02 (1.33–3.07)	3.34 (2.52–4.43)	7.07 (3.94–12.67)	249.43 (164.44–378.22)	338.96 (255.33–449.87)	558.11 (310.78–999.76)	+249	+124
20–24	2.24 (1.48–3.40)	3.70 (2.78–4.91)	7.82 (4.36–14.00)	288.19 (189.85–437.31)	360.06 (271.02–478.21)	830.20 (462.40–1486.46)	+249	+188
25–29	2.38 (1.57–3.60)	3.92 (2.96–5.20)	8.30 (4.63–14.83)	249.27 (164.43–377.73)	478.13 (360.75–633.51)	1071.19 (597.59–1914.55)	+249	+329
30–34	2.42 (1.60–3.65)	3.99 (3.02–5.26)	8.43 (4.71–15.04)	205.79 (136.04–311.18)	506.37 (383.40–668.57)	837.98 (468.43–1494.68)	+249	+307
35–39	2.37 (1.57–3.58)	3.91 (2.97–5.16)	8.28 (4.63–14.76)	208.14 (137.67–314.55)	404.40 (306.56–533.29)	788.56 (441.14–1405.54)	+249	+278
40–44	2.27 (1.50–3.43)	3.75 (2.84–4.94)	7.93 (4.44–14.14)	142.97 (94.56–216.08)	313.54 (237.73–413.40)	944.93 (528.69–1684.21)	+249	+560
45–49	2.14 (1.42–3.24)	3.54 (2.68–4.66)	7.49 (4.19–13.34)	104.03 (68.84–157.15)	302.62 (229.67–398.62)	924.81 (517.65–1647.94)	+249	+789
50–54	2.02 (1.33–3.06)	3.33 (2.52–4.41)	7.05 (3.94–12.59)	91.45 (60.35–138.54)	201.68 (152.44–266.74)	700.89 (391.51–1251.68)	+249	+666
55–59	1.93 (1.27–2.94)	3.19 (2.39–4.25)	6.75 (3.76–12.10)	78.90 (51.83–120.07)	145.26 (109.05–193.44)	532.83 (296.59–954.95)	+249	+575
60–64	1.89 (1.24–2.88)	3.12 (2.34–4.17)	6.61 (3.67–11.86)	61.42 (40.29–93.58)	127.25 (95.33–169.81)	514.26 (285.89–922.86)	+249	+737
65–69	1.88 (1.23–2.87)	3.11 (2.32–4.16)	6.58 (3.65–11.83)	46.17 (30.22–70.50)	105.87 (79.04–141.76)	336.59 (186.75–605.22)	+250	+629
70–74	1.89 (1.23–2.92)	3.12 (2.30–4.24)	6.62 (3.64–11.98)	35.68 (23.13–55.01)	74.30 (54.68–100.93)	223.30 (122.97–404.51)	+250	+526
75–79	1.91 (1.21–3.01)	3.15 (2.25–4.41)	6.68 (3.62–12.29)	21.30 (13.52–33.53)	45.91 (32.82–64.19)	161.31 (87.46–296.77)	+250	+657
80+ years	1.94 (1.18–3.19)	3.20 (2.15–4.75)	6.77 (3.53–12.95)	13.83 (8.41–22.77)	38.51 (25.92–57.26)	151.44 (79.00–289.61)	+250	+995
Total	1.99 (1.31–3.02)	3.36 (2.53–4.47)	7.15 (3.98–12.82)	2299.87 (1514.15–3492.29)	4269.45 (3213.85–5671.27)	9843.44 (5479.62–17638.67)	+259	+328

**Figure 4:**
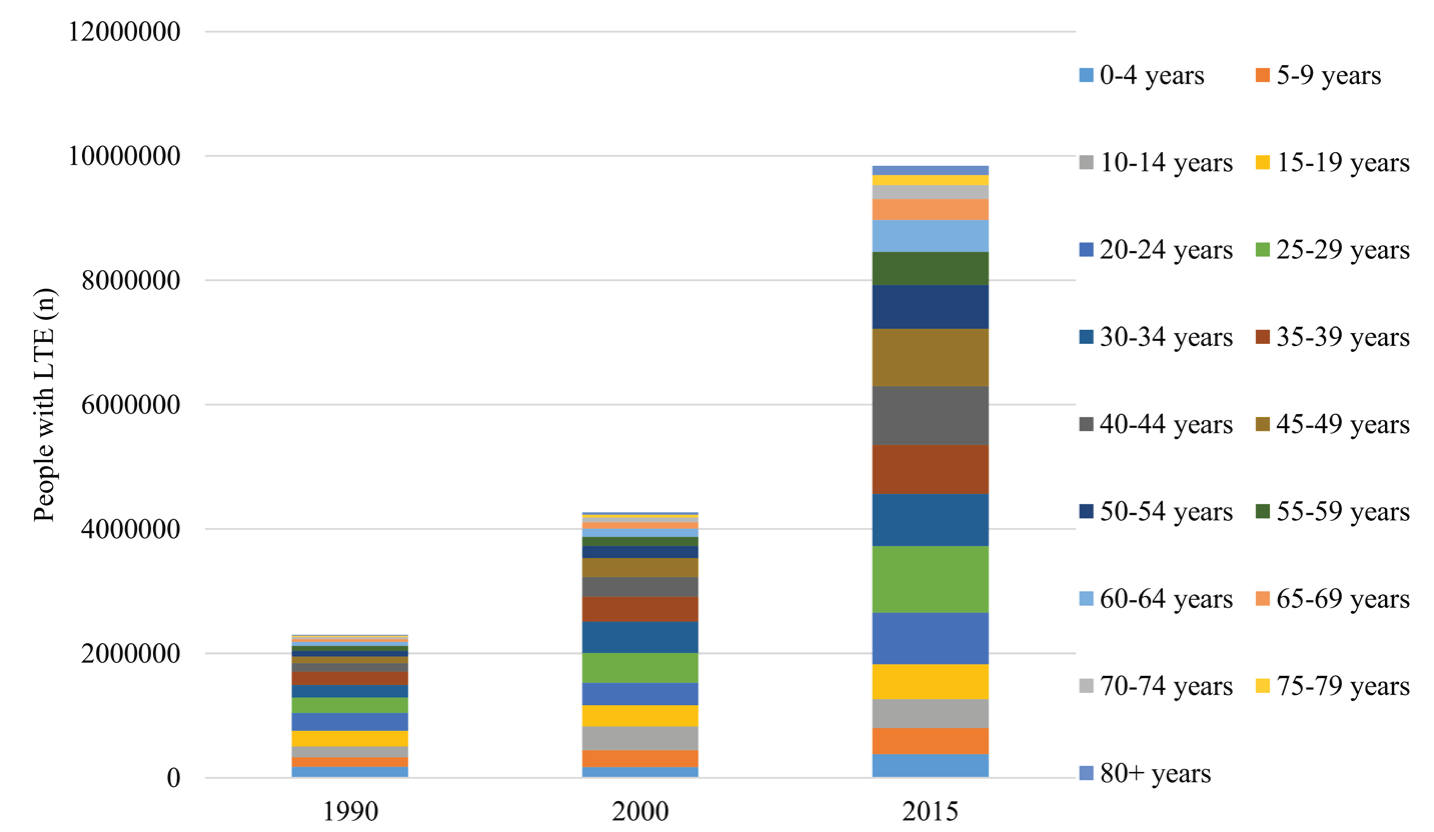
Estimated numbers of people with lifetime epilepsy (LTE) in China by year and age group.

The estimated number of individuals with LTE in China was 2.30 million (95% CI = 1.51–3.49) in 1990, and 9.84 million (95% CI = 5.48–17.64) in 2015, which implied an overall increase of 328% throughout this period (**Table 2** and **Figure 4**). The most significant increase of LTE cases was observed among people aged 40 years and above, where the rates of change were all above 500%, and the highest was noted in people aged 80+ years. Most LTE cases were in individuals aged 20–24 years in 1990, and then shifted to the group of 25–29 years in 2015.

### Estimates of AE prevalence and number of cases in China

**Figure 5:**
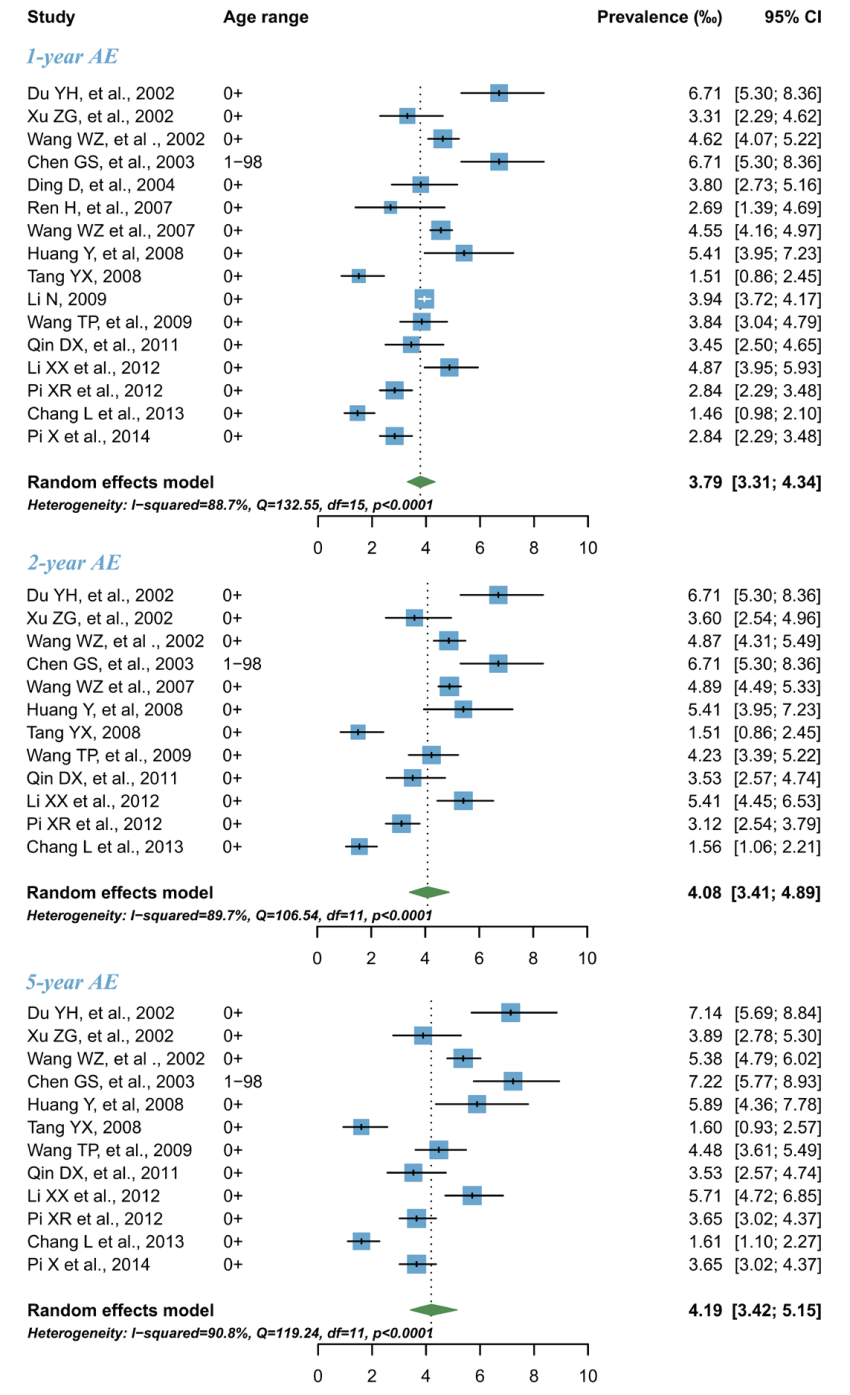
Pooled prevalence of 1–year, 2–year and 5–year active epilepsy (AE) in China by random–effects meta–analysis.

As shown in **Figure 5**, the pooled 1–year AE prevalence across all time periods was 3.79‰ (95% CI = 3.31–4.34), and the 2–year and 5–year AE prevalence were slightly higher, as expected, amounting to 4.08‰ (95% CI = 3.41–4.89) and 4.19‰ (95% CI = 3.42–5.15) respectively. When these estimates are applied to the Chinese population size in the year 2015, the numbers of individuals with 1–year, 2–year and 5–year AE cases were estimated to 5.22 million (95% CI = 4.55–5.97), 5.61 million (95% CI = 4.69–6.73) and 5.77 million (95% CI = 4.71–7.09), accounting for more than half of all the contemporary LTE cases.

## DISCUSSION

This study describes a comprehensive estimate of the prevalence of epilepsy in China from 1990 to 2015. Although this is the second attempt to summarize the available data on epilepsy prevalence in China, our study is the first (to the best of our knowledge) that provides robust estimates of LTE prevalence across the entire age range. It also reveals the prevalence of 1–year, 2–year and 5–year AE simultaneously in Chinese population. This is of particular clinical and public health interest, because individuals with AE may benefit more from treatment compared to those with LTE [10,33]. In addition, we also conducted a temporal analysis of LTE burden in China from 1990 to 2015. This innovative exploration is of potential value in studying the effect of previous national antiepileptic programs. Significant strengths of this study include exhaustive search strategy, attempts to reduce bias by rigorous study selection procedure, parallel review and extraction of data.

In the current study, LTE was defined as “at least two unprovoked (or reflex) seizures, except for febrile convulsion and seizure induced by acute brain diseases at some point during lifetime”. Our study revealed LTE prevalence of 1.99‰ in 1990 and 7.15‰ in 2015 in China. Our estimate of LTE prevalence for the year 1990 was in line with the estimates presented in the previous systematic review, which reported a prevalence of 1.84‰ for the period of 1986–1990, and 3.58‰ for the period of 1991–1995 respectively [19]. Our estimate of LTE prevalence in 2015 was higher than their estimate for the period from 2006–2010 (7.15 ‰ vs 6.62‰), which is plausible given that a dramatic increase of LTE prevalence was encountered in our study.

When we perform comparisons to the global estimate of LTE prevalence, our estimate of LTE prevalence for the year 2015 (7.15‰) was even lower than the lower band of the estimated range in LMICs (8.75‰, 95% CI = 7.23–10.59]) but equal to the upper band of that in HICs; 5.18‰, 95% CI = 3.75–7.15) [16,24,38]. Similarly, our estimate of 5–year AE (4.19‰) is much more in line with the estimate in developed countries (4.9‰, 95% CI = 2.3–10.3), but less than in developing countries [10]. Despite the higher rates of spontaneous remission of epilepsy cases, given that fact that epilepsy is still a highly stigmatising disorder in China, any concealment could make these estimates even higher [26,39].

Epilepsy is known to affect people of all ages, though more frequently affecting young people [40]. The estimates in our study indicated that the LTE prevalence peaked at individuals aged 30–34 years. This finding confirms the statement made in previous studies that the epilepsy is generally a disease of the young [23]. The peak of this disorder in young age could partly be explained by the accumulation of early–onset epilepsy cases. It is estimated that the incidence of epilepsy is the highest in young children and elderly, forming a characteristic U–shape [21,41]. In addition, individuals with epilepsy may at a higher risk of premature death. Two previous studies reported that the risk of premature death in individuals with epilepsy is 3–5 times higher than in general population, and especially among the young [42,43]. According to the survival effects theory, it is plausible that the lifetime prevalence of epilepsy showed a decreasing trend after the age of 30–34 years in our study. This phenomenon has also been seen in many other neurological diseases [44].

The gender difference is another interesting topic in epilepsy research, both relevant to the public health and clinical research. Previous investigations reported slightly higher prevalence estimates of epilepsy in males than in females, which was probably attributable to the inherent differences in healthy brain development between genders and marked social effects on disease risks and courses [19,45,46]. However, in our analysis, the difference of LTE prevalence between sexes was not statistically significant. This finding is in contrast with the previous systematic review of epilepsy prevalence in China, but in line with the synthesised results in Asia, Latin America and Europe and the whole globe [21,23,24,47]. These inconsistencies with other studies may be a result of the different proportional contribution of epilepsy subtypes to the included case series. According to previous evidence, localization–related symptomatic epilepsy was more prevalent in males, and cryptogenic localization–related epilepsy was more prevalent in females [46,48].

In this study, we confirmed the hypothesis that the prevalence of LTE was continually increasing by presenting a positive temporal trend between 1990 and 2015. The prevalence of LTE is determined by the incidence rate at which new cases arise and the mortality rate [10]. Given the increased life expectancy and rapid ageing process in China during the two decades, it is reasonable to expect a dramatic increase rate of LTE prevalence through cumulative effects [49,50].

In the current study, we developed an estimate of the 1–year, 2–year and 5–year AE prevalence in China, which provides a basis for future studies, especially in the research of treatment gap for epilepsy in China [33,34]. However, because of the scarcity of studies that reported age– or gender–specific AE prevalence, we were not able to undertake some more detailed studies. Compared to the global 1–year AE prevalence (4.6‰ in 2000 and 4.5‰ in 2000) [15] in the Campaign Against Epilepsy Demonstration Project conducted in rural China, our study revealed a much lower 1–year AE prevalence of 3.79‰ in general population.

The magnitude of epilepsy burden in China, estimated in this study, represents a huge and significant health and socioeconomic burden [34,51]. With proper antiepileptic medication, up to 70% of epileptic seizures can be well controlled [52,53]. However, previous studies suggested that more than half of the individuals with epilepsy in China had never been treated with appropriate antiepileptic medicines [15,39]. Many barriers may contribute to this situation. In China, due to the stigma–attached nature of the diagnosis of epilepsy, individuals with epilepsy are generally socially isolated and suffer in silence [15,39]. Individuals with epilepsy are more likely to be under–educated or under–employed. A lack of knowledge about the nature of epilepsy and treatment may also influence the patients’ personal health–seeking behaviours and compliance [15]. In addition, most individuals with epilepsy may be economically disadvantaged, which makes the free provision of antiepileptic medicines critically important for the management of epilepsy. However, this goal has not been universally achieved across the whole country, especially in resource–poor areas [52]. To make the situation worse, the lack of electroencephalogram and neuroimaging equipment, and personnel with neurologic expertise, may severely restrict the diagnosis of patients to a large extent.

Our study also had several potential limitations. First, our estimate of epilepsy prevalence was based on cross–sectional studies in the community. However, because of the uncertainty regarding each case definition, and the ratio between sensitivity and specificity of the diagnostic tools used, it has already been pointed that cross–sectional assessment may considerably underestimate the prevalence [44,54,55]. In addition, the initial suspicion on epilepsy in most of our included studies was established through the use of questionnaires or interviews. Although it was later also confirmed by neurologists, this investigatory approach may still be problematic due to recall bias and a high proportion of concealment [15,39]. Second, large variations were observed between the studies included in our systematic analysis. We tried to minimise these variations through strict inclusion and exclusion criteria. Still, variations in study methods, approaches to sampling, diagnostic criteria, availability of or access to appropriate treatment led to a considerable variation. Third, our modelled estimates were based on a limited number of covariates that were available in our included studies. Future attempts at evidence synthesis should include more of these covariates, such as the subtype of epilepsy, level of economic development and treatment gap. With these limitations in mind, the estimates presented in this study need to be interpreted cautiously.

The results of our meta–analysis have both academic and public health implications. An immense deficit in epidemiologic data regarding age–specific AE prevalence and LTE prevalence in China was identified. In particular, new studies should also adopt appropriate methods to reduce the variation in reported prevalence. Other important contributors include identifying subtypes of epilepsy and the current barriers in the society and health care systems. From the public health perspective, it is well–documented that most epilepsy cases can be prevented by effective measurements. Common preventable causes in children include infectious diseases, prenatal and perinatal central nervous system damage [2,16]. Birth asphyxia and febrile seizures are also well–documented risk factors for epilepsy. Programs targeted at reducing birth asphyxia and timely treatment of febrile convulsions can also contribute to the reduction of epilepsy burden [56,57]. In adults, the most prevalent causes of epilepsy include head injury, intracranial infection and cerebrovascular diseases, which account for 88.5% of all the adult–onset epilepsy cases [58,59], highlighting the importance of preventing injury.

## CONCLUSIONS

Our study provides a comprehensive evidence synthesis of LTE and AE prevalence in China to date. LTE prevalence is the highest in individuals aged 30–34 years. The burden of LTE in China has increased dramatically between 1990 and 2015, when the prevalence of LTE has more than doubled, and the number of people with LTE more than tripled. The large amount of AE cases in China calls for optimal management and treatment. More high–quality epidemiological studies on LTE and AE prevalence are still needed.
