# Supplementary material for: Prevalence of epilepsy in China between 1990 and 2015: A systematic review and meta–analysis
Source: J Glob Health. 2017 Dec 22;7(2):020706. doi: 10.7189/jogh.07-020706 (PMC5737100; doi:10.7189/jogh.07-020706)
Supplement: Online Supplementary Document [file jogh-07-020706-s001.pdf]

Online Supplementary Document

Song et al. Prevalence of epilepsy in China between 1990 and 2015: A systematic review and meta-analysis

J Glob Health 2017;7:020706

Table S1. Search strategies in CNKI, Wanfang, VIP and PubMed

Database	Access date	Subject category	Sub-database	Search terms	Publication date	Search method
**CNKI**	04/02/2016	Medicine & Public Health	Journal, Featured journal, Doctoral dissertation, Master dissertation, Domestic conferences, International conferences	(SU % 'dianxian' + 'epilepsy') AND (SU % 'fabinglv' + 'fashenglv' + 'huanbinglv'+ 'lihuanlv' + 'xianhuanlv'+ 'siwanglv' + 'bingsilv'+ 'liuxing' + 'fudan'+ 'xiankuangdiaocha'+ 'xiankuangyanjiu')	01/01/1990 - 04/02/2016	Comprehensive search: subject, title, keywords and abstract
**Wanfang**	03/02/2016	Not applicable	Journal articles, Dissertations, Conference articles	(subject: (dianxian) + subject: (epilepsy))* (subject: (fabinglv) + subject: (fashenglv) + subject: (huanbinglv)+ subject: (lihuanlv) + subject: (xianhuanlv) + subject: (siwanglv)+ subject: (bingsilv) + subject: (liuxing) + subject: (fudan)+ subject: (xiankuangdiaocha) + subject: (xiankuangyanjiu))	1990-2016	Comprehensive search: subject (including title, keywords and abstract)
**VIP**	04/02/2016	Medicine & Public Health	All journals	(M=(dianxian+epilepsy)+R=(dianxian+epilepsy))*(M=(fabinglv +fashenglv+huanbinglv+lihuanlv+xianhuanlv+siwanglv+bingsilv+liuxing+fudan+xiankuangdiaocha+xiankuangyanjiu)+R=(fabinglv+fashenglv+huanbinglv+lihuanlv+xianhuanlv+siwanglv+bingsilv+liuxing+fudan+xiankuangdiaocha+xiankuangyanjiu))	1990-2016	Comprehensive search: title, keywords and abstract
**PubMed**	05/02/2016	Not applicable	Not applicable	((epilepsy) AND (China OR Chinese) AND (inciden* OR prevalen* OR morbidity OR mortality)) AND ("1990/01/01"[Date - Publication] : "2016/02/03"[Date - Publication])	01/01/1990 - 03/02/2016	Comprehensive search: all fields

Table S2. Detailed characteristics of the included studies (n=39)

Study	Province	Setting	Screening tool	Diagnosis of epilepsy	Age range	Sample size	LTE prevalence (‰)	1-year AE prevalence (‰)	2-year AE prevalence (‰)	5-year AE prevalence (‰)
Wang DS, et al. (2002)	Heilongjiang	Rural	WHO screening questionnaire	By neurologists	0+	10157	7.97	-	-	-
Huang MS,et al. (2002)	Shanghai	Both	Screening questionnaire	By trained investigators	0+	48628	3.11	-	-	-
Zhang C, et al. (1990)	Beijing	Urban	Screening questionnaire	By neurologists	0-14	6837	3.07	-	-	-
Liu XH, et al. (1990)	Shaanxi	Mixed	WHO screening questionnaire	By neurologists	0+	161163	0.89	-	-	-
Zhong XX (1991)	Guangdong	Mixed	WHO screening questionnaire	By neurologists	0+	176165	0.49	-	-	-
Tang H, et al. (1991)	Jiangsu, Anhui, Zhejiang, Jiangxi, Fujian	Mixed	Screening questionnaire	By neurologists	20+	540580	0.28	-	-	-
He Y, et al. (1991)	Yunnan	Urban	Screening questionnaire	By neurologists	0+	67891	0.57	-	-	-
Tao T, et al. (1994)	Guizhou	Mixed	Screening questionnaire	By trained investigators	0+	9173	2.18	-	-	-
Da GW (1997)	Tibet	Rural	WHO screening questionnaire	By trained physicians	0+	31423	2.42	-	-	-
Zhang AH, et al. (1997)	Hunan	Rural	WHO screening questionnaire	By trained physicians	0+	806254	1.70	-	-	-
Zhou DM, et al. (2000)	Anhui	Mixed	Screening questionnaire	By neurologists	0+	9098	1.32	-	-	-

Hong Z, et al. (2000)	Zhejiang	Rural	Screening questionnaire based on ILAE diagnosis	By neurologists	0+	88515	1.94	-	-	-
Qin DX, et al. (2011)	Henan	Rural	WHO screening questionnaire	By neurologists	0+	12457	4.74	3.45	3.53	3.53
Du YH, et al. (2002)	Ningxia	Rural	WHO and ICBERG screening questionnaire	By neurologists	0+	11630	8.47	6.71	6.71	7.14
Xu ZG, et al. (2002)	Shanxi	Rural	WHO screening questionnaire	By neurologists	0+	10273	5.84	3.31	3.60	3.89
Wang WZ, et al. (2002)	Heilongjian, Ningxia, Henan, Shanxi, Jiangsu	Rural	WHO screening questionnaire	By neurologists	0+	55616	6.96	4.62	4.87	5.38
Chen GS, et al. (2003)	Ningxia	Rural	WHO screening questionnaire	By trained physicians	1-98	11630	8.47	6.71	6.71	7.22
Ding D, et al. (2004)	Shanghai	Rural	Epilepsy epidemiological Survey form made by BNI	By neurologists	0+	10777	6.03	3.80	-	-
Wei G (2004)	Tibet	Both	Screening survey form for epilepsy, self-designed epilepsy survey form	By neurologists	3+	6968	7.18	-	-	-
Zhang HZ, et al. (2005)	Jiangsu	Rural	Epilepsy epidemiological Survey form made by BNI	By neurologists	0+	11118	7.83	-	-	-
Ren H, et al. (2007)	Yunnan	Rural	WHO screening questionnaire	By trained physicians	0+	4465	3.81	2.69	-	-
Huang Y, et al (2008)	Ningxia	Rural	WHO screening questionnaire	By neurologists	0+	8316	8.30	5.41	5.41	5.89
Tang YX (2008)	Yunnan	Rural	WHO screening questionnaire	By neurologists	0+	10597	1.79	1.51	1.51	1.60
Li N (2009)	Jilin	Rural	Screening questionnaire	By neurologists	0+	310402	-	3.94	-	-
Wang TP, et al. (2009)	Shanxi	Rural	WHO and ICBERG screening questionnaire	By neurologists	0+	10273	5.84	3.21 in 2000; 4.47 in 2004	3.60 in 2000; 4.86 in 2004	3.89 in 2000; 5.06 in 2004

Wu SP et al. (1993)	Hunan, Sichuan, Guangdong, Heilongjian, Niangxia	Both	WHO screening questionnaire	By neurologists	0+	108306	4.26	-	-	-
Li JH et al. (1994)	Yunnan	Mixed	CMA standard 1985	NS	0+	10390	1.35	-	-	-
Wang H et al. (1994)	Hunan	Rural	Screening questionnaire	By neurologists	0+	43330	1.20	-	-	-
Zhan C et al. (1999)	Sichuan	Mixed	Screening questionnaire	By neurologists	0+	43992	2.94	-	-	-
Wang WZ et al. (2007)	Heilongjian, Ningxia, Henan, Shanxi, Jiangsu	Rural	WHO screening questionnaire	NS	0+	51644	6.20	4.62 in 2000; 4.47 in 2004	4.87 in 2000; 4.92 in 2004	-
Zhao YC et al. (2011)	Shanghai	Urban	Screening questionnaire based on ILAE diagnosis	By neurologists	0-103	106573	4.81	-	-	-
Li XX et al. (2012)	Hebei	Mixed	WHO and ICBERG screening questionnaire	By neurologists	0+	20138	6.85	4.87	5.41	5.71
Pi XR et al. (2012)	Hunan	Rural	WHO and ICBERG screening questionnaire	By neurologists	0+	32059	4.46	2.84	3.12	3.65
Chang L et al. (2013)	Jiangsu	Urban	Screening questionnaire based on ILAE diagnosis	By trained physicians	0+	19860	1.71	1.46	1.56	1.61
Guo MH et al. (2014)	Shandong	Rural	WHO and ICBERG screening questionnaire	By trained physicians and neurologists	0+	251492	1.33	-	-	-
Liu AY et al. (2015)	Yunnan	Mixed	Present State Examination	By neurologists	15+	4934	0.81	-	-	-
Qi S (2015)	Jiangxi	Rural	Screening questionnaire based on ILAE diagnosis	By trained physicians	0+	7632	4.59	-	-	-
Zhao YH et al. (2010)	Tibet	Rural	NS	By neurologists	0+	7996	22.51	-	-	-

Pi X et al. (2014)	Hunan	Rural	WHO screening questionnaire	By neurologists	0+	32059	-	2.84	-	3.65

* *Note: WHO=World Health Organization; ICBERG= International Community-based Epilepsy Research Group; ILAE= International League Against Epilepsy; CMA= Chinese Medical Association; BNI= Beijing Neurosurgical Institute; "-"= data not available.*

Table S3. Univariate meta-regression models of the relationship between different variables and the LTE prevalence

Variable	Number of data points	Number of studies	β	95 % CI	P value
Intercept	275	37	-5.797	[-6.112]-[-5.482]	<0.001
Age	275	37	-5.482	0.001- 0.004	0.012
Gender-Male	173	17	-0.020	[-0.107]- 0.0676	0.659
Study year	275	37	0.053	0.020- 0.085	0.001

Note: coefficients (*β*) *represent log odds ratios (ORs).*
